# Undisturbed *Posidonia oceanica* meadows maintain the epiphytic bacterial community in different environments

**DOI:** 10.1007/s11356-023-28968-x

**Published:** 2023-08-07

**Authors:** Alice Rotini, Chiara Conte, Gidon Winters, Marlen I. Vasquez, Luciana Migliore

**Affiliations:** 1 ISPRA Istituto Superiore per la Protezione e la Ricerca Ambientale, Via Vitaliano Brancati, 48, 00144 Rome, Italy; 2 Department of Biology, Laboratory of Ecology and Ecotoxicology, University of Rome Tor Vergata, 00133 Rome, Italy; 3 Dead Sea and Arava Science Center (DSASC), Masada National Park, 86910 Masada, Israel; 4 Eilat Campus, Ben-Gurion University of the Negev, Hatmarim Blv., 8855630 Eilat, Israel; 5 Department of Chemical Engineering, Cyprus University of Technology, 30 Archbishop Kyprianos Str.t, 3036 Limassol, Cyprus; 6 European University of Technology, 30 Archbishop Kyprianos Str.t, 3036 Limassol, Cyprus; 7 eCampus University, Via Isimbardi 10, 22060 Novedrate, CO Italy

**Keywords:** Seagrass ecology, Seagrass holobiont, Ecological descriptors, *Posidonia oceanica*, Total phenols, Photosynthetic pigments, Marine bacteria, Cyprus

## Abstract

Seagrasses harbour different and rich epiphytic bacterial communities. These microbes may establish intimate and symbiotic relationships with the seagrass plants and change according to host species, environmental conditions, and/or ecophysiological status of their seagrass host. Although *Posidonia oceanica* is one of the most studied seagrasses in the world, and bacteria associated with seagrasses have been studied for over a decade, *P. oceanica*’s microbiome remains hitherto little explored. Here, we applied 16S rRNA amplicon sequencing to explore the microbiome associated with the leaves of *P. oceanica* growing in two geomorphologically different meadows (e.g. depth, substrate, and turbidity) within the Limassol Bay (Cyprus). The morphometric (leaf area, meadow density) and biochemical (pigments, total phenols) descriptors highlighted the healthy conditions of both meadows. The leaf-associated bacterial communities showed similar structure and composition in the two sites; core microbiota members were dominated by bacteria belonging to the Thalassospiraceae, Microtrichaceae, Enterobacteriaceae, Saprospiraceae, and Hyphomonadaceae families. This analogy, even under different geomorphological conditions, suggest that in the absence of disturbances, *P. oceanica* maintains characteristic-associated bacterial communities. This study provides a baseline for the knowledge of the *P. oceanica* microbiome and further supports its use as a putative seagrass descriptor.

**Supplementary Information:**

The online version contains supplementary material available at 10.1007/s11356-023-28968-x.

## Introduction

*Posidonia oceanica* is a keystone seagrass species endemic to the Mediterranean Sea (Hartog and Kuo 2007) where it is considered a biodiversity hotspot. *P. oceanica* meadows are also the foundation of one of the most characteristic habitats of the Mediterranean Sea (Boudouresque 2004; Boudouresque et al. 2006; Larkum et al. 2006). Its three-dimensional structure creates spawning grounds, nurseries, or permanent habitats for many species, supporting a complex community, which colonises the above- and below-ground plant compartment (including *matte*) (Bellan-Santini et al. 1986; Borg et al. 2006). Furthermore, *P. oceanica* meadows act as a carbon sink (blue carbon) mitigating climate change (Pedersen et al. 2011; Pergent-Martini et al. 2021), one of the most valuable ecosystem services for our times (Apostolaki et al. 2011; Marx et al. 2021). However, *P. oceanica* is sensitive to increasing temperatures, low and high salinities (Boudouresque et al. 2006; Jordà et al. 2012), pollution, and other anthropic pressures (Boudouresque et al. 2006; Jordà et al. 2012; Pazzaglia et al. 2020). Since its ecological status is tightly related to the quality of its surrounding environment, *P. oceanica* is considered a bioindicator (Montefalcone 2009) and a target of specific conservation and protection measures. At the international level, *P. oceanica* is protected under the Bern and the Barcelona Conventions, and *P. oceanica* meadows fall among the habitats of priority interest included in the European Union’s Habitat Directive (92/43/CEE). Moreover, the Marine Strategy Framework Directive (MSFD, 2008/56/EC) selected *P. oceanica* as a representative species of the angiosperm quality elements for the Mediterranean marine environment.

*P. oceanica* is a K-strategist, long-lived seagrass species characterised by the very slow growth of its plagiotropic and orthotropic rhizomes (a few centimetres per year; Boudouresque et al. 2006). The species is able to successfully colonise sandy bottoms as well as bare rocky substrates (Den Hartog 1970; Boudouresque and Meinesz 1982; Hemminga and Duarte 2000). Recent studies underlined the influence of the substrate type on the success of *P. oceanica* seed recruitment and tolerance to hydrodynamic regimes (Alagna et al. 2015; Badalamenti et al. 2015; Montefalcone et al. 2016; Ruju et al. 2018; Zenone et al. 2022).

Seagrasses host a variety of epiphytic organisms, from eukaryotic micro and macroalgae, invertebrates, fungi, viruses, to prokaryotics (Ettinger and Eisen 2019; Mejia et al. 2016; Supaphon et al. 2017; Tarquinio et al. 2021), which may strongly influence the plants’ physiology (Brodersen et al. 2018; Conte et al. 2021a; Crump et al. 2018; Tarquinio et al. 2019; Ugarelli et al. 2017). Hence, each shoot may be considered a network of interactions in which the host and all associated organisms living in/on its tissues establish transient or lasting different relationships, resulting in a complex functional unit, the so-called ‘holobiont’ (*sensu* Zilber-Rosenberg and Rosenberg 2008). The role of the epiphytic bacterial community and its potential effects on the seagrass ecophysiology has been drawing attention in recent years. It may enhance nutrients availability and uptake (e.g. Garcias-Bonet et al. 2016; Tarquinio et al. 2018; Welsh 2000) and increase seagrass growth by producing growth hormone-like compounds (Celdrán et al. 2012; Conte et al. 2021a; Crump et al. 2018; Tarquinio et al. 2019; Ugarelli et al. 2017; Zilber-Rosenberg and Rosenberg 2008). It can contribute to the host’s defence by producing antimicrobial compounds (Egan et al. 2013; Longford et al. 2019) and by degrading phytotoxic compounds, like H_2_S and ethanol (Brodersen et al. 2018; Crump et al. 2018; Holmer et al. 2001). In turn, seagrasses provide these epiphytic communities chemically different colonizable surfaces and labile or recalcitrant organic matter (Brodersen et al. 2018; Crump et al. 2018; Martin et al. 2018; Tarquinio et al. 2019; Ugarelli et al. 2017).

Due to the high bacterial turnover, the holobiont is potentially a dynamic entity in which the microbial partner’s composition may change over time and environmental conditions, including changes in host ecophysiology (Mejia et al. 2016; Rotini et al. 2017; Rotini et al. 2020; Tarquinio et al. 2019). The rapid changes in the microbial community structure and composition can facilitate the holobiont’s adaptation to the continuous and unpredictable changes in environmental conditions (Carrier and Reitzel 2017; Duarte et al. 2018); on the other hand, the disruption of the host microbial associations may lead to host pathologic conditions (Bang et al. 2018; Egan et al. 2013; Longford et al. 2019; Martin et al. 2020; Pitlik and Koren 2017; Sullivan et al. 2018). As a consequence, host biology and ecology remain intimately connected with their microbial partners (Mejia et al. 2016; Brodersen et al. 2018). Therefore, identifying the structure and composition of the epiphyte communities is fundamental for improving our understanding of seagrass ecology and establishing more efficient ecosystem management strategies.

Studies on *P. oceanica* epiphytic bacteria have been performed mainly by culture-dependent approaches (García-Martínez et al. 2009; Marco-Noales et al. 2006); these studies suggested a link between the associated bacterial community and the meadow decline (Carrier and Reitzel 2017; García-Martínez et al. 2009) or the enhancement of leaf growth (Garcias-Bonet et al. 2016). Studies by metagenomic approaches are relatively few and have mainly focused on the roots (Garcias-Bonet et al. 2016; Lehnen et al. 2016; Kohn et al. 2020; Conte et al. 2021a, 2021b). They reported a high N_2_ fixation and sulphate reduction rate associated with *P. oceanica* roots. Only a few recent studies focused on the leaf epiphytic bacteria; they underline the potential mutual microbes-seagrass relationship and the variation of seagrass associated with the host condition. Kohn et al. (2020) found an increase in the diversity of the leaf-associated bacterial community with increasing leaf age. In the Cyprus Limassol Bay, in a residual patch of *P. oceanica* in the proximity of Limassol port, Conte et al. (2021b) found a functional link between plant descriptors and the hosted microbial community. In that study, *P. oceanica* showed a very high total phenol content, indicating a deteriorated environmental condition and a high relative abundance of bacterial families belonging to the Bulkholderiales order. These bacteria are known degraders of complex C-compounds, including phenols (Nešvera et al. 2015), and their presence indicates how leaf physiology might affect the epiphytic bacteria composition.

The present study is aimed at deepening the knowledge about the bacterial communities associated with *P. oceanica* leaves along with the plants’ ecophysiological descriptors to explore their potential use for the seagrass health status assessment. Specific objectives of the study were (i) to characterise the associated bacterial communities in meadows growing in two sites around Limassol (Cyprus) and (ii) to evaluate if differences in habitat features (depth, substrate type, turbidity) may affect and change the associated bacterial communities. To this aim, the structure and taxonomic composition of the leaf epiphytic bacterial communities were analysed by 16S rDNA gene analysis; to link these microbial communities with the ecophyisological status of their host, we also analysed morphometric (leaf area, meadow density) and biochemical (pigments, total phenols) descriptors.

## Materials and methods

### Study area and sampling

**Fig. 1:**
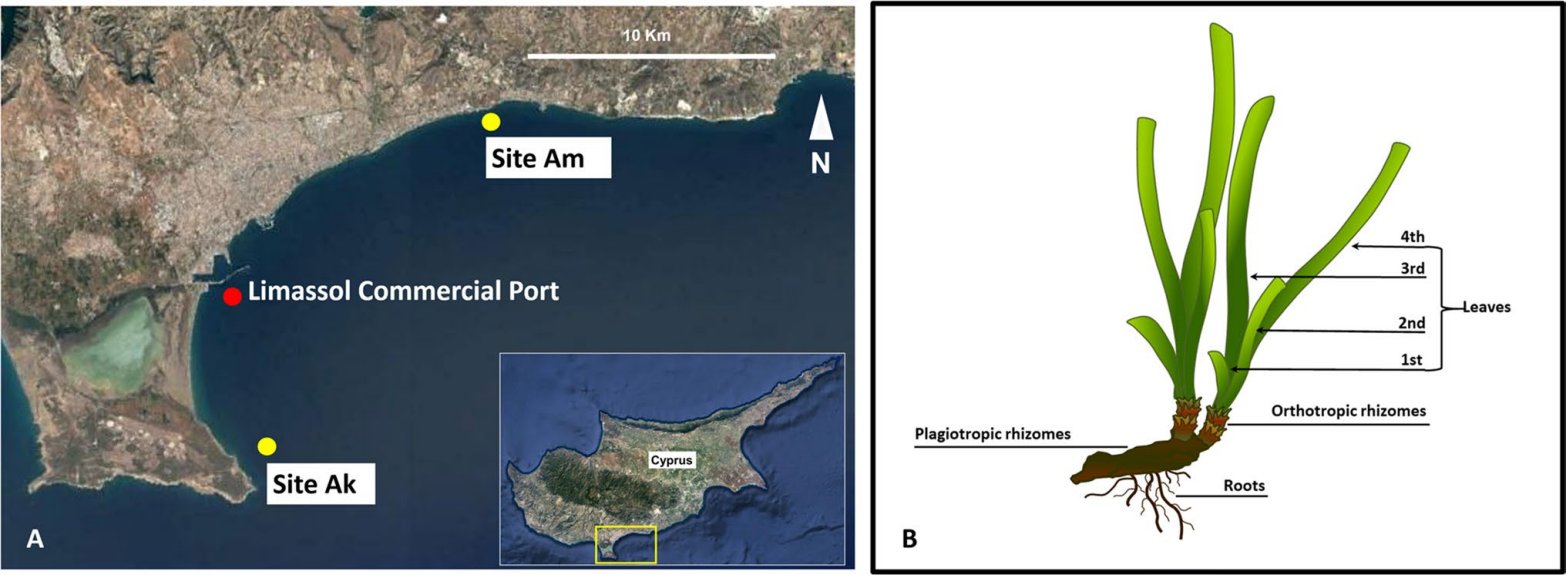
**A** The two sampling sites in the Limassol-Akrotiri Bay (Cyprus Island, eastern Mediterranean Sea): Akrotiri, Ak, and Amathus, Am (yellow dots; map source: Google Earth, 2020); **B** schematic representation of *Posidonia oceanica* shoot composed by rhizome with its roots and leaves (from A. Rotini PhD Thesis, 2011); leaf numbering is indicated from the youngest to the oldest

Sampling activities were conducted in December 2017 by SCUBA-diving in two *Posidonia oceanica* meadows located in the region of the Limassol-Akrotiri Bay (Fig. 1A), which showed different habitat features. Site Ak: Akrotiri-Royal Air Force base (within the Public Access Area (34° 34.83′ N, 33° 2.235′ E) is generally considered a pristine area. Here, the *P. oceanica* meadow occurs at 7–8 m depth and about 200 m from the shore. It was thick and stood mostly on hard substrate, with some patches growing on soft bottoms. Site Am: Ancient Port of Amathus (34° 42.36′ N, 33° 08.38′ E), a protected archaeological submerged site. The patchy *P. oceanica* meadow occurs at 1.5–2 m depth and about 50 m from the shore; it stands on a hard substrate (ancient ruins) and is the only meadow left at shallow depth (< 5 m) within the Limassol Bay.

Seagrass samples were collected on a soft bottom in Ak and on a rocky substrate in Am; as the study was aimed to evaluate if, in ‘healthy’ meadows, habitat features such as depth or substrate may affect/change the seagrass associated bacterial communities, as a first approach, one single sampling event was considered the best option in order to limit the effect of other possible sources of variability. Furthermore, in a framework of ecological ethics, only leaves in three replicates were sampled, and not rhizomes which would entail harming the integrity of the meadows. The second-last leaf in order of emergence within the shoot (a.k.a. second leaf) was chosen for all the analyses (Fig. 1B): it is big enough to allow all the analyses and young enough, not to be affected by senescence processes (Kohn et al. 2020; Iqbal et al. 2023).

For plant descriptor analyses, *P. oceanica* leaves were haphazardly sampled within each site: the second leaf was cut right above the rhizome from 30 different ramets, at a minimum of 2 m distance from each other, avoiding sampling at the meadow edge. Similarly, for bacterial analyses, the second leaf was cut right above the rhizome from 3 different and randomly chosen shoots, at a minimum of 5 m distance from each other, in each site. The second leaf was chosen in order to study an established bacterial biofilm, avoiding the possible impact of leaf senescence. Each leaf was stored separately underwater, in a ziplock bag to keep the bacterial communities as much as possible unaffected and to separate different replicates.

Three replicates of seawater samples were collected right above the plants (1 L). Three replicates of bulk sediment samples were collected by a mini corer (2.5 cm in diameter and 5 cm in depth). All samples were stored in a cooler until they arrived at the laboratory of the Cyprus University of Technology (within about 30 min) and then kept at 4 °C under dark until sample processing (within 12 h from the sampling). A CTD probe measured temperature, salinity, and pH during plant sampling (Table 1).

**Table 1:** Environmental parameter values recorded during the sampling

Sampling site	Temperature* (°C)	Salinity* (ppt)	pH*	Visibility ^†^ (m)	Sea current ^†^
Akrotiri	20.89	39	8.27	15	None
Amathus	20.82	39	8.24	5-6	Strong

*CTD measurements; ^†^operators’ observations

### Plant and meadow descriptors

#### Shoot density and biometry

The density of *P. oceanica* meadow was assessed by counting underwater 3 times the shoots inside a quadrate (20 × 20 cm); shoot density was reported as the number of shoots m^−2^. All the leaves collected (30) were digitally scanned (Cannon Lide 120) and analysed by the ImageJ platform (version 1.47; Schneider et al. 2012) to calculate the leaf surface area (cm^2^).

#### Biochemical analyses

Biochemical analyses were performed on 15 leaves per site, as briefly described below.

*Photosynthetic pigments* (chlorophyll *a* and chlorophyll *b*, total carotenoids) were extracted in duplicate from leaf tissues (250 mg fresh weight, each), grounded in liquid N_2_ using a mortar and pestle, in 2.5 ml of methanol (4 °C, overnight) according to Wellburn (1994), modified by Rotini et al. (2013a). Quantification of pigments in the extracts was performed with a spectrophotometer (JENWAY 7315, Staffordshire, UK) by measuring the absorption at 470, 652, 665, and 750 nm, and concentrations of these pigments (as mg g^−1^ of fresh weight) were calculated according to Wellburn (1994).

*Phenolic compounds* were extracted in duplicate from leaf tissue (100 mg fresh weight, each), grounded in liquid N_2_ using a mortar and pestle, in 4 ml of 0.1 N HCl (4 °C, overnight) and quantified according to Migliore et al. (2007). The quantification of total phenols was performed in spectrophotometry at 724 nm; concentrations were expressed as chlorogenic acid equivalents (mg) per gramme of plant material (fresh weight, FW).

### Bacterial community

At each site, bacterial communities were collected separately from three second *P. oceanica* leaves, sediment, and bottom seawater samples. In the laboratory, under sterile conditions, each leaf was carefully and repeatedly gently scraped on both sides with a sterile blade sprinkling with a pipette with 2 ml of washing solution, to wash away the biofilm (washing solution: 200 mM Tris–HCl pH 8, 10 mM EDTA, and 0.24% Triton X-100; Kadivar and Stapleton 2003); the solution was then centrifuged (20′, 5000*g*), and the pellet stored in 2 ml of transport solution (transport solution: Tris 10 mM, EDTA 50 mM; Kadivar and Stapleton 2003) to preserve it, as reported in Mejia et al. (2016). Three sediment samples were stirred and 2 g of mixed sediment per replicate were stored, each submerged in transport solution until DNA extraction. Three seawater samples (1 L per replicate) were collected underwater just above the meadows. In the laboratory, each litre was filtered by a vacuum pump equipped with a sterile 0.2 μm Whatman® membrane filter sterile apparatus. The filters were stored, each submerged in transport solution until DNA extraction.

The bacterial metagenomic DNA was extracted by the Power Soil® DNA isolation kit (Mo Bio, Carlsbad, CA, USA), according to the manufacturer’s instructions. The 16S rRNA gene was amplified by PCR with the universal primers Com1 (forward, 5′-AGCAGCCGCGGTAATAC-3′) and Com2 (reverse, 5′-CGTCAATTCCTTTGAGTTT-3′) that amplify the hypervariable region V3-V4) as reported in Mejia et al. (2016) and Schmalenberger et al. (2001); the amplified DNA was then purified by Gel/PCR Fragment Extraction Kit (GeneAid, Taiwan). The pure DNA extracts were sent to Molecular Research LP (MR DNA Shallowater, Texas, USA) for sequencing by an NGS Illumina MiSeq platform. The raw paired-end sequences were analysed by the Quantitative Insights Into Microbial Ecology (QIIME 2.10; Bolyen et al. 2019) pipeline. The sequences were demultiplexed, quality and chimaera checked, and filtered by the DADA2 QIIME2 plugin (Callahan et al. 2016). As a total, 2258 ASVs (Amplicon Sequence Variants, i.e. each inferred single DNA sequence recovered from a high-throughput analysis of 16S rDNA genes) were identified, with a frequency of 512,299 reads. Taxonomic identification of the 16S rRNA gene sequences was performed using a Naive Bayes classifier trained with the SILVA 138 SSU database (Quast et al. 2012). ASVs classified as chloroplasts or mitochondria were discarded from the dataset. The rarefaction curves, built to evaluate differences and efficiency in the sampling effort, confirmed that the sequencing coverage was good (see Supplementary Fig. S1). The dataset was normalised at the common depth of 15,567 sequences per sample, the lowest number of sequences in the dataset (sample *P. oceanica* leaves, site A, replicate #1). Although this imposed a low sequence number, it allowed keeping three biological replicates of each sample type in the dataset. The final dataset (cleaned from chloroplasts and mitochondria sequences and normalised) was composed of a total of 2187 ASVs, used to perform the downstream analyses (Tab. S1). Statistical analyses were performed within QIIME (Bolyen et al. 2019) or PAST 4.05 (Hammer et al. 2001).

This Targeted Locus Study project has been deposited at GenBank as Bioproject PRJNA916897.

### Statistical analyses

Differences in leaf area (*n* = 31), meadow density (*n* = 3) or pigments-total phenols contents (*n* = 12) were evaluated by Student’s *t*-test.

Bacterial diversity within samples (α-diversity) was estimated using Shannon-Wiener Index (PAST 4.05; Hammer et al. 2001; Legendre and Legendre 1998). Pearson correlation (QIIME2, alpha correlation plugin; Bolyen et al. 2019; Pearson 1895) was used to test the possible relationship between seagrass leaf bacterial α-diversity and leaf biochemical parameters. Stratified permutational multivariate analysis of variance (Adonis R Vegan function; Oksanen et al. 2020) with Bray-Curtis dissimilarity was used to evaluate significant differences of β-diversity in the whole dataset using sites and matrices as a source of variance. These data were visualised by PCoA (QIIME2; Bolyen et al. 2019; Halko et al. 2011). To detect finer differences, each sample type was compared by one-way ANOSIM with Bray-Curtis dissimilarity (PAST 4.05; Legendre and Legendre 2012; Hammer et al. 2001).

Venn diagrams were built to visualise shared and unique ASVs in leaf-associated bacterial communities and to identify the bacterial core (the shared component; https://bioinformatics.psb.ugent.be/webtools/Venn/). Bar plots were used to visualise the bacterial core agglomerated at the family level. The analysis of the composition of microbiomes (ANCOM; Mandal et al. 2015) was used on the dataset agglomerated at the family level to detect significant differences in the distribution of the bacterial communities associated with seagrass leaves. The thorough list of the leaf-associated bacteria in each replicate, agglomerated at the family level, was used to build the heatmap (PAST 4.05, Hammer et al. 2001, visualised in Excel).

## Results and discussion

The ecological status of *Posidonia oceanica* was evaluated in meadows from two sites of the Cyprus Island, where environmental conditions are different (§ 2.1; Fig. 1A). To this end, morpho-physiological seagrass descriptors together with the composition of the associated bacterial communities were analysed in each site.

*Morpho-physiological descriptors* are widely used to identify the health status of seagrass plants (i.e. Winters et al. 2011; Rotini et al. 2013a; Schubert et al. 2015; Collier et al. 2009; Ceccherelli et al. 2018; Beca-Carretero et al. 2019). In this study, a comparable shoot density was observed in the two sites (Table 2), although slightly higher in Akrotiri than in Amathus. Differences were not significant (Student’s *t*-test, *P* > 0.05) and the average density values (< 400 shoots/m^2^) account for dense meadows in both sites (classification of Giraud 1997, modified by Pergent et al. 1995). Also, leaf total phenol content did not differ between the two sites and showed quite low values. Again, this indicates a healthy and comparable ecological status of the two meadows, as the phenol content is a seagrass descriptor is known to increase under stressed conditions (Dumay et al. 2004; Migliore et al. 2007; Rotini et al. 2013a, 2013b; Ceccherelli et al. 2018; Mannino and Micheli 2020; Conte et al. 2023). Plants from both Amathus and Akrotiri sites were found to contain half the total phenol content of their counterparts growing in the polluted area of the Limassol port (Conte et al. 2021b). Furthermore, the concentrations found in this study are comparable to those already recorded in meadows from pristine sites (Fresi et al. 2004; Costa et al. 2015). Some differences were found in leaf biometry and photosynthetic pigment content, both showing higher values in Amathus (Am) than in Akrotiri (Ak), even though Am is the shallowest site. These differences probably depend on the different local light regimes (high turbidity and strong currents in Amathus; see Table 1), but both these light regimes did not represent a stressing condition for plants, being comparable in the two sites the value of chlorophyll *a*/*b* ratio, a marker of light stress (Casazza and Mazzella 2002). Overall, the morpho-physiological seagrass descriptors depict a similar and balanced plant ecophyisological status of both *P. oceanica* meadows, despite the geomorphological differences between sites (e.g. depth, substrate, and turbidity), but in agreement with the good ecological conditions of both sites (e.g. lack of pollutants or anthropic pressure).

**Table 2:** Seagrass meadow and plant descriptors in the two sampling sites: Ak (Akrotiri) and Am (Amathus). Meadow density (as n° shoots/*m*^2^ ± SE, *n* = 3), mean leaf area (as mm^2^ ± SE, *n* = 30) and mean total phenols and photosynthetic pigment contents (as mg/g FW ± SE, *n* = 15) are reported, along with the results of comparisons between sites by Student’s *t*-test (DF, *N*, *t*, and *P* values)

Seagrass parameter	Site		Comparison between sites (Student’s *t*-test)			
	Ak	Am	DF	*N*	*t*	*P* value
Meadow density (shoots m^−2^)	569.8 ± 38.2	410.4 ± 31.5	4	3	− 2.5801	n.s
Leaf area (cm^−2^)	2475 ± 105.6	3668 ± 210.5	60	31	− 3.9976	*P* < 0.001
Total phenols (mg g^−1^ of FW)	21.98 ± 3.34	20.82 ± 4.49	22	12	0.2083	n.s.
Chlorophyll *a* (mg g^−1^ of FW)	0.260 ± 0.015	0.470 ± 0.040	28	15	4.1981	*P* < 0.001
Chlorophyll *b* (mg g^−1^ of FW)	0.156 ± 0.010	0.292 ± 0.028	28	15	4.5118	*P* < 0.001
Carotenoids (mg g^−1^ of FW)	0.139 ± 0.007	0.214 ± 0.016	28	15	4.1423	*P* < 0.001
Total Chl/Car	2.97 ± 0.08	3.53 ± 0.17	28	15	2.9837	*P* < 0.01
Chl *a*/ Chl *b*	1.67 ± 0.04	1.61 ± 0.03	28	15	− 1.379	n.s.

*Chl a*, chlorophyll *a*; *Chl b*, chlorophyll *b*; *Car*, total carotenoids; *FW*, fresh weight

**Fig. 2:**
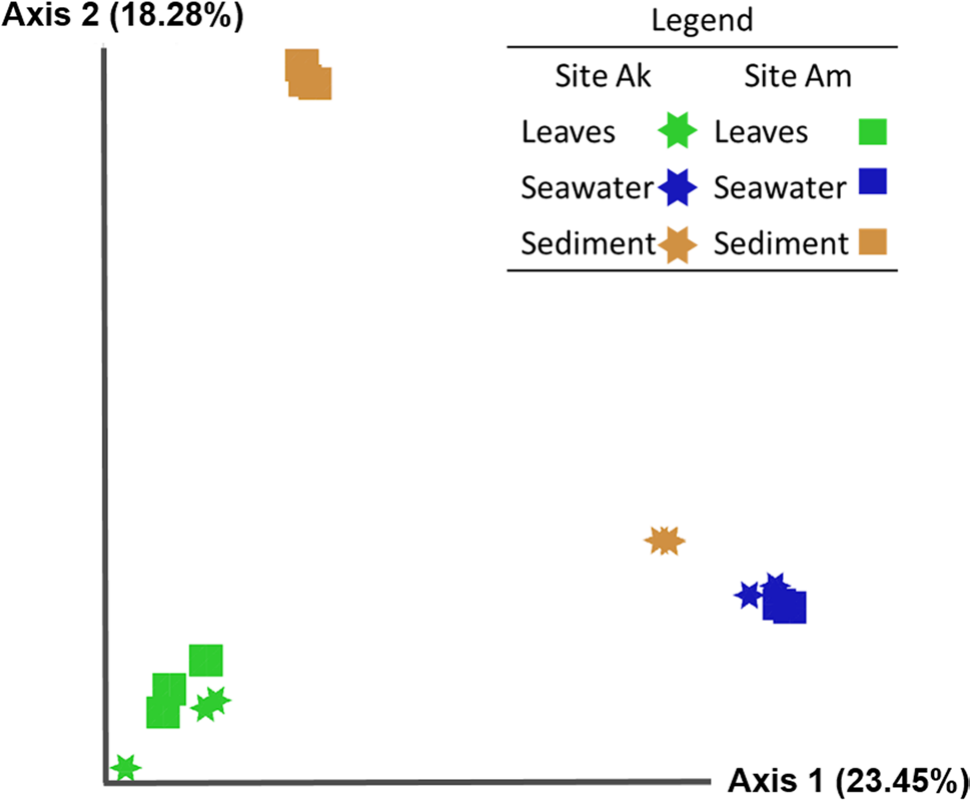
Principal Coordinate Analysis (PCoA) of the bacterial communities (whole dataset), based on Bray-Curtis dissimilarity metrics, showing the distance among bacterial communities sampled from seagrass leaf, seawater and sediment samples, at each site (Ak and Am)

*Seagrass and associated bacterial communities* are considered a dynamic unity, the seagrass *holobiont*, and the structure and composition of the bacterial communities change with the environmental conditions and/or plant traits, helping plants to cope with environmental changes (Conte et al. 2021a). Because of this tight relationship, the alteration of the bacterial communities may be marker and/or responsible for damaged conditions of the host (Bang et al. 2018; Egan et al. 2013; Longford et al. 2019; Martin et al. 2020; Pitlik and Koren 2017; Sullivan et al. 2018). In the two meadows of Cyprus, consistently with results from plant descriptors, a comparable pattern was found in the bacterial communities associated with *P. oceanica* plants: the α-diversity of the bacterial community showed comparable values in the two sites. Generally, the bacterial diversity of both seawater and *P. oceanica* leaves was higher in Ak, while sediment bacterial α-diversity was higher in Am (Table 3). As expected, an overall significant difference in the bacterial communities’ structure and composition (β-diversity) was found among sample types (i.e. seawater, sediment, and leaves) and sites (ADONIS, *P* < 0.05; Fig. 2). No significant differences in β-diversity were found between the bacterial community of the different sample types between sites, except for the sediment (ANOSIM, *P* < 0.05); this was expected, due to the intrinsic differences in substrate types: a soft bottom in Ak and a rocky substrate in Am (Vasquez M., personal comm.), with different characteristics and available microenvironments in the two colonizable substrates. By comparing leaf-associated and seawater or sediment bacteria, no differences were found between leaf-associated and seawater bacterial community in both sites (β-diversity; ANOSIM, *P >* 0.05), and this was expected because the seawater free-living bacteria and those associated with suspended particles represent the bacterial microbial pool, and the suspended particles are considered the main source of leaf colonisers (Fahimipour et al. 2017; Iqbal et al. 2021, 2023).

**Table 3:** Comparison of the bacterial community α-diversity (Shannon-Wiener Index) between the two sites (*Ak*, Akrotiri; *Am*, Amathus). Values represent the means of three replicated samples in each site

Site	Shannon-Wiener Index (± SD; *n* = 3)		
	*P. oceanica* leaves	Seawater	Sediment
Ak	3.21 ± 0.90	4.53 ± 0.06	3.64 ± 0.21
Am	3.02 ± 1.06	3.51 ± 1.20	3.18 ± 0.23

**Fig. 3:**
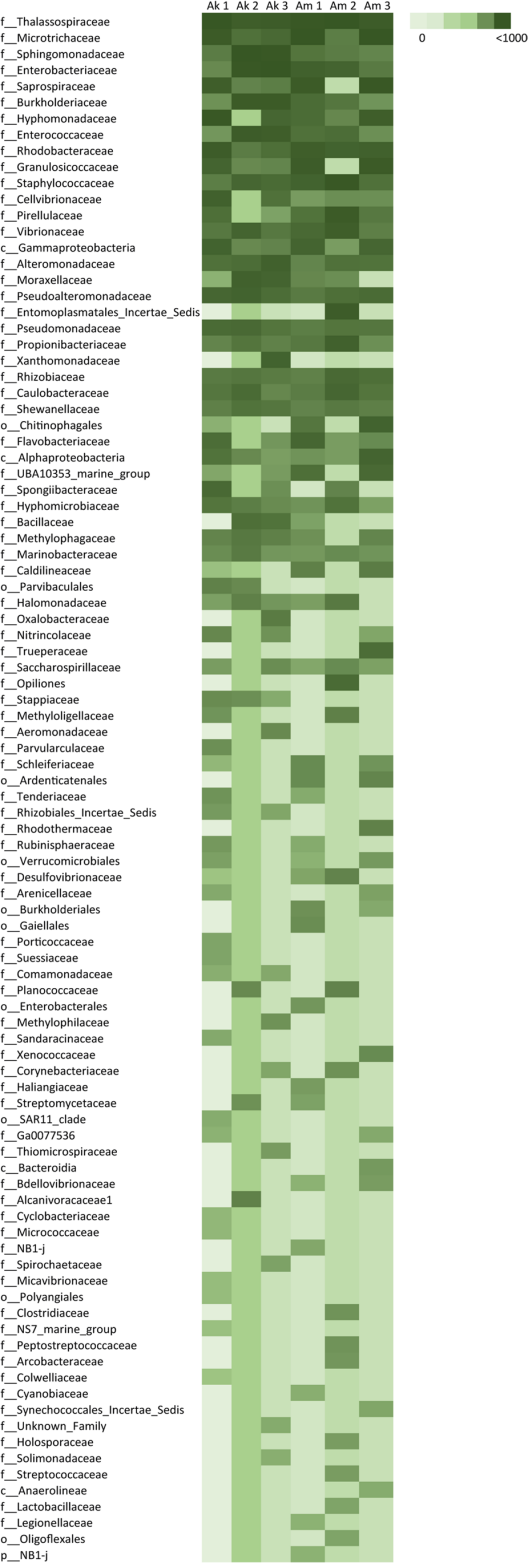
Distribution of the bacterial families among the replicates of *Posidonia oceanica* leaves from Akrotiri (Ak1, Ak2, Ak3) and Amathus (Am1, Am2, Am3), displayed as a heatmap. Colour accounts for log_10_ frequency (light colour = low frequency, dark colour = high frequency)

**Fig. 4:**
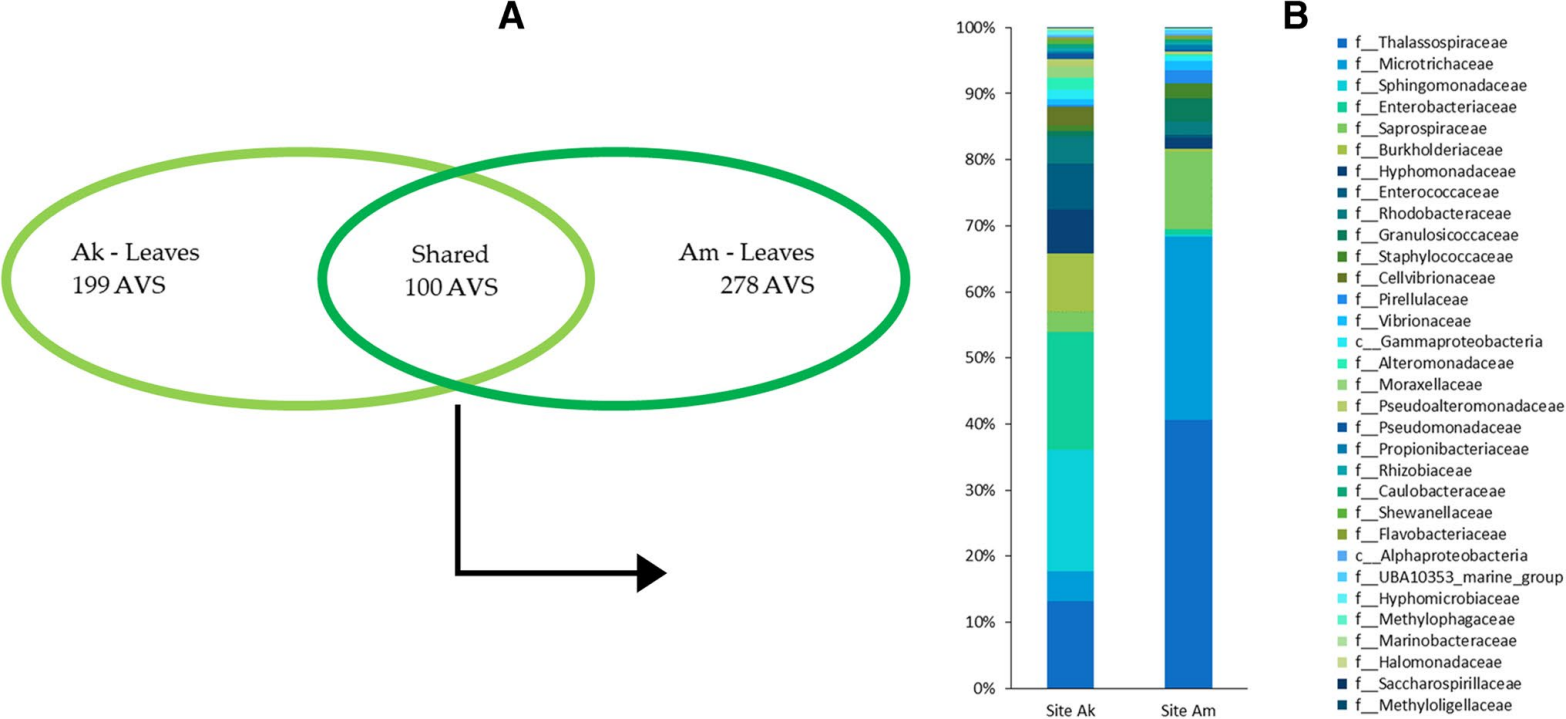
**A** Venn diagram showing the distribution of unique and shared Amplicon Sequence Variants (ASVs) associated with *Posidonia oceanica* leaves in the two sites, Akrotiri (Ak) and Amathus (Am), and **B** bar plot showing the relative abundances of the core bacterial families in each site

The taxonomic composition of the bacterial communities found in different sample types was evaluated as Amplicon Sequence Variants (ASVs), using the normalised dataset agglomerated at the family level. The leaf-associated bacterial communities evaluated at family level displayed similar structure and composition (Fig. 3). Leaf-associated bacterial communities showed a common dominant component in the two sites (Fig. 4), i.e. a high number of shared ASVs: 100 shared ASVs were found in the communities from the two sites, accounting for 81% (37,827 reads) and 78% (36,659 reads) ASVs from Ak and Am, respectively. These shared ASVs represent the bacterial core of both communities. The unique ASVs found in each leaf community accounted for 19% (8874 reads) and 21% (10,042 reads) in Am and Ak, respectively, representing the environmental ‘fingerprint’, i.e. the peculiar bacteria linked to the specific conditions of each site, the site-specific bacterial colonisers (Fig. 4A). In both sites, the *P. oceanica* bacterial core (i.e. the core micobiome) was composed of Thalassospiraceae, Microtrichaceae, Enterobacteriaceae, Saprospiraceae, and Hyphomonadaceae families (Fig. 4B). Among them, some are known as marine biofilm-forming bacteria such as Thalassospiraceae, an aerobic chemoorganotrophic bacterial family with the ability to reduce nitrate (Imhoff and Wiese 2014); others, like Microtrichaceae and Hyphomonadaceae, are potentially involved in leaf nitrate supply (Abraham and Rohde 2014; Korlević et al. 2021; Szitenberg et al. 2022). Alongside, abundant families were Saprospiraceae, known to break down complex organic carbon (McIlroy and Nielsen 2014), and Enterobacteriaceae, widespread ammonifying bacteria (Rehr and Klemme 1989). Thus, the dominant component of the bacterial core is likely involved in pivotal basic processes for the host plant as, among others, nitrogen cycling. Slight differences were found in the unique components, with very few bacterial families uniquely associated with the plants of each site: in Ak 7, unique bacterial families were found at low percentages (< 3%; Cellvibrionaceae, Alteromonadaceae, Moraxellaceae, Pseudomonadaceae, Xanthomonadaceae, Shewanellaceae, and Spongiibacteraceae), and in Am 4, unique bacterial families were found at low percentages (< 3%), three classified at order and one at class level (ASV that have not been recognised further in the taxonomic rank). These bacteria belong to Pirellulaceae, Propionibacteriaceae, Flavobacteriaceae, Hyphomicrobiaceae, Entomoplasmatales, Chitinophagales, Alphaproteobacteria, and two other unknown families. The composition comparison of leaf-associated microbiomes (ANCOM) did not highlight significant differences in the bacterial families’ distribution. These results, in the holobiont perspective, confirm that under comparable good environmental conditions—in spite of geomorphological differences—leaf-associated bacterial communities are similar and involved in plants in basic processes; hence, they suggest to be related to the healthy status of seagrass plants in both sites.

Results from the present study combined with those from a recent study performed in the same area (Limassol-Akrotiri Bay), on the same dates and with the same technical protocols (Conte et al. 2021b), further support the functional link between seagrass plant traits and their associated bacterial communities. In this study, the comparable structure and composition of leaf-associated *P. oceanica* bacterial community superimposes with the similar eco-physiological status of plants in the two meadows, notwithstanding the different habitat features of the two sites. Conversely, in the area of the Limassol port, investigated by Conte et al. (2021b), which is considered polluted and located in between the two undisturbed meadows from this study (as indicated by the red dot in Fig. 1A), *P. oceanica* plants displayed ecophysiological signs of stress, i.e. a very high total phenol content in leaves (twice the amount of Amathus and Akrotiri plants) and associated bacterial communities that were completely different from the associated bacterial communities on undisturbed meadows. In the Limassol port, the plants’ stressed condition was mirrored by an ad hoc composition of the epiphytic bacterial community: *P. oceanica* leaves hosted bacteria of Bulkholderiales order, at relatively high abundance (Conte et al. 2021b); this order includes families able to degrade phenols (Nešvera et al. 2015). Again, the high concentrations of total phenols and the presence of Bulkholderiales in the Limassol port plants supported the tight relationship between seagrasses and their leaf-associated bacterial communities.

## Conclusion

In conclusion, this study provides new insights into the knowledge of the bacterial communities associated with the iconic seagrass *P. oceanica*, which until now has been very little explored.

The ecophysiological seagrass descriptors applied here alongside our molecular work, depicted a similar and good plant conservation status in the two sites, despite the differences in habitat features (substrate type, depth, turbidity). The similar seagrass ecophysiology between the two different sites, resulted in a similar recruitment of bacterial communities, confirming that ecophysiological conditions, rather than habitat features, shape the seagrass associated epiphytic microbial community. As already observed by Conte et al. (2021b), seagrass showed ‘elective affinities’ with their associated bacteria, further supporting the tight and functional relationship plant/bacteria and the bacterial involvement in plant homeostasis.

Furthermore, the two sites, in spite of geomorphological differences, can be considered pristine sites with comparable good environmental conditions: in the holobiont perspective, the occurrence of the same bacterial core strengthens the assumption of their functional role, supporting the use of associated bacteria as an important source of ecological information and a putative seagrass health descriptor.

## Supplementary information

ESM 1 (DOCX 48 kb)

ESM 2 (XLSX 155 kb)

## Data Availability

The sequences have been deposited in GenBank as Targeted Locus Sequences under the BioProject ID PRJNA916897.
