# Supplementary figures and images for: Undisturbed Posidonia oceanica meadows maintain the epiphytic bacterial community in different environments
Source: Environ Sci Pollut Res Int. 2023 Aug 7;30(42):95464–74. doi: 10.1007/s11356-023-28968-x (PMC10482771; doi:10.1007/s11356-023-28968-x)

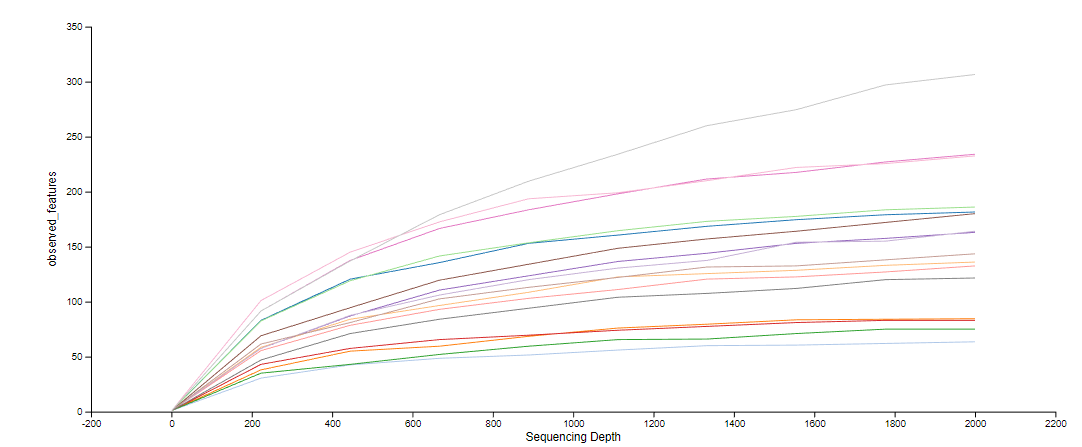


Figure S1 Rarefaction curves

Supplement: Supplementary file 1 — (DOCX 48 kb) [file 11356_2023_28968_MOESM1_ESM.docx]
